# Hormone replacement therapy and risk of epithelial ovarian cancer

**DOI:** 10.1038/sj.bjc.6690731

**Published:** 1999-10

**Authors:** D M Purdie, C J Bain, V Siskind, P Russell, N F Hacker, B G Ward, M A Quinn, A C Green

**Affiliations:** Department of Social and Preventive Medicine, The University of Queensland, Medical School, Herston, Queensland, 4006, Australia; Department of Anatomical Pathology, Royal Prince Alfred Hospital, Camperdown, New South Wales, 2050, Australia; Gynaecological Cancer Centre, Royal Hospital for Women, Paddington, 188 Oxford Street, Paddington, New South Wales, 2021, Australia;; Department of Obstetrics and Gynaecology, The University of Queensland, Royal Brisbane Hospital, Herston, Queensland, 4029, Australia; Gynaecologic Oncology/Dysplasia Unit, The Royal Women's Hospital, 132 Grattan Street, Carlton, Victoria, 3053, Australia; Queensland Institute of Medical Research, Royal Brisbane Hospital, Herston, Queensland, 4029, Australia

**Keywords:** ovarian neoplasms, case–control study, oestrogen replacement therapy

## Abstract

It has been suggested that oestrogen replacement therapy is associated with risk of epithelial ovarian cancer of the endometrioid type. Using data from an Australian population-based case–control study, the relation between unopposed oestrogen replacement therapy and epithelial ovarian cancer, both overall and according to histological type, was examined. A total of 793 eligible incident cases of epithelial ovarian cancer diagnosed from 1990 to 1993 among women living in Queensland, New South Wales and Victoria were identified. These were compared with 855 eligible female controls selected at random from the electoral roll, stratified by age and geographic region. Trained interviewers administered standard questionnaires to obtain detailed reproductive and contraceptive histories, as well as details about hormone replacement therapy and pelvic operations. No clear associations were observed between use of hormone replacement therapy overall and risk of ovarian cancer. Unopposed oestrogen replacement therapy was, however, associated with a significant increase in risk of endometrioid or clear cell epithelial ovarian tumours (odds ratio (OR) 2.56; 95% confidence interval (CI) 1.32–4.94). In addition, the risk associated with oestrogen replacement therapy was much larger in women with an intact genital tract (OR 3.00; 95% Cl 1.54–5.85) than in those with a history of either hysterectomy or tubal ligation. Post-menopausal oestrogen replacement therapy may, therefore, be a risk factor associated with endometrioid and clear cell tumours in particular. Additionally, the risk may be increased predominantly in women with an intact genital tract. These associations could reflect a possible role of endometriosis in the development of endometrioid or clear cell ovarian tumours. © 1999 Cancer Research Campaign


*British Journal of Cancer* (1999) **81**(3), 559–563
© 1999 Cancer Research Campaign
Article no. bjoc.1999.0731

### Hormone replacement therapy and risk of epithelial ovarian cancer

**DM Purdie[1], CJ Bain[1], V Siskind[1], P Russell[2], NF Hacker[3], BG Ward[4], MA Quinn[5] and AC Green[6]**

[1]Department of Social and Preventive Medicine, The University of Queensland, Medical School, Herston Road, Herston, Queensland 4006, Australia; [2]Department of Anatomical Pathology, Royal Prince Alfred Hospital, Missenden Road, Camperdown, New South Wales 2050, Australia; [3]Gynaecological Cancer Centre, Royal Hospital for Women, 188 Oxford Street, Paddington, New South Wales 2021, Australia; [4]Department of Obstetrics and Gynaecology, The University of Queensland, Royal Brisbane Hospital, Herston, Queensland 4029, Australia; [5]Gynaecologic Oncology/Dysplasia Unit, The Royal Women's Hospital, 132 Grattan Street, Carlton, Victoria 3053, Australia; [6]Queensland Institute of Medical Research, Royal Brisbane Hospital, Herston, Queensland 4029, Australia

**Summary** It has been suggested that oestrogen replacement therapy is associated with risk of epithelial ovarian cancer of the endometrioid type. Using data from an Australian population-based case–control study, the relation between unopposed oestrogen replacement therapy and epithelial ovarian cancer, both overall and according to histological type, was examined. A total of 793 eligible incident cases of epithelial ovarian cancer diagnosed from 1990 to 1993 among women living in Queensland, New South Wales and Victoria were identified. These were compared with 855 eligible female controls selected at random from the electoral roll, stratified by age and geographic region. Trained interviewers administered standard questionnaires to obtain detailed reproductive and contraceptive histories, as well as details about hormone replacement therapy and pelvic operations. No clear associations were observed between use of hormone replacement therapy overall and risk of ovarian cancer. Unopposed oestrogen replacement therapy was, however, associated with a significant increase in risk of endometrioid or clear cell epithelial ovarian tumours (odds ratio (OR) 2.56; 95% confidence interval (CI) 1.32–4.94). In addition, the risk associated with oestrogen replacement therapy was much larger in women with an intact genital tract (OR 3.00; 95% Cl 1.54–5.85) than in those with a history of either hysterectomy or tubal ligation. Post-menopausal oestrogen replacement therapy may, therefore, be a risk factor associated with endometrioid and clear cell tumours in particular. Additionally, the risk may be increased predominantly in women with an intact genital tract. These associations could reflect a possible role of endometriosis in the development of endometrioid or clear cell ovarian tumours. © 1999 Cancer Research Campaign

**Keywords**: ovarian neoplasms; case–control study; oestrogen replacement therapy

Unopposed oestrogen replacement therapy (ORT) is an established risk factor for endometrial adenocarcinoma (Grady et al, 1995). The relation between hormone replacement therapy (HRT) and ovarian cancer remains uncertain, however. Since cancer of the ovary shares some risk factors with cancer of the endometrium, such as low parity (Whittemore et al, 1992) and obesity (Purdie et al, 1995), post-menopausal oestrogen therapy may also have a role in its aetiology. This may particularly be so for the endometrioid epithelial form, which is histologically similar to adenocarcinoma of the endometrium (Russel, 1994). Some studies have shown unopposed oestrogen therapy for at least 1 year to be associated with an increase in risk of endometrioid tumours predominantly (La Vecchia et al, 1982; Weiss et al, 1982; Cramer et al, 1983; Risch, 1996) while others have suggested either no effect (Booth et al, 1989; Kaufman et al, 1989; Whittemore et al, 1992) or an inverse association (Hartge et al, 1988; Hempling et al, 1997) between oestrogens and this type of ovarian cancer. Some of these studies, however, have involved small numbers of exposed cases (La Vecchia et al, 1982; Cramer et al, 1983; Kaufman et al, 1989), have not distinguished unopposed oestrogen from oestrogen given in combination with progestogen (Booth et al, 1989; Hempling et al, 1997), or have used hospital controls (Hartge et al, 1988; Booth et al, 1989; Kaufman et al, 1989; Hempling et al, 1997) with possible overestimation of general exposure rates (Rodriguez et al, 1995). Hence, the issue is currently unresolved.

We report here the results of a large Australian case–control study in which the relation between HRT, and in particular unopposed ORT, and epithelial ovarian cancer, both overall and among histological types, was explored. We also examined any potential modification of the effect of ORT on ovarian cancer risk by tubal sterilization and hysterectomy.

*Received 5 October 1998*
*Revised 19 March 1999*
*Accepted 24 March 1999*

*Correspondence to:* DM Purdie, Department of Social and Preventive Medicine, 4th Floor – North Wing, Diamantina House, Princess Alexandra Hospital, Ipswich Road, Woolloongabba, Queensland 4102, Australia

### METHODS

Histologically confirmed incident cases of primary epithelial ovarian cancer registered in major gynaecological-oncology treatment centres in three Australian states were ascertained. Cases diagnosed in 1991 and 1992 in New South Wales and Victoria, and from August 1990 to the end of 1993 in Queensland were recruited. The histological types of all cancers of women included in the study were reviewed by an independent gynaecological pathologist in each state, as described in detail elsewhere (Purdie

**Table 1** Ever use of hormone replacement therapy among all cases of epithelial ovarian cancer and controls with crude and adjusted odds ratios

| HRT use | Cases (*n* = 793)[a] | Controls (*n* = 855)[a] | Crude OR (95% CI) | Adjusted OR[b] (95% CI) |
|---|---|---|---|---|
| None[c] | 83.5% | 82.6% | 1.00 | 1.00 |
| Any HRT | 16.5% | 17.4% | 0.94 (0.72–1.21) | 1.20 (0.90–1.60) |
| Unopposed oestrogen | 8.6% | 8.8% | 0.97 (0.68–1.36) | 1.27 (0.86–1.88) |
| Unopposed progestogen | 1.8% | 1.3% | 1.36 (0.61–3.01) | 2.18 (0.91–5.20) |
| Oestrogen and progestogen in combination | 4.9% | 5.4% | 0.90 (0.58–1.40) | 1.34 (0.83–2.17) |
| Not known | 2.9% | 2.7% | 1.07 (0.59–1.92) | 1.05 (0.55–2.01) |

[a]The combined total number from treatment groups is greater than the total number of HRT users as 13 cases and six controls reported use of different hormone treatments at different times. [b]ORs adjusted for age, education, area of residence, BMI, hysterectomy, tubal sterilization, talc use in perineal region, smoking status, duration of OCP use, parity and a family history of breast or ovarian cancer. [c]Reference category for all forms of HRT.

et al, 1995). Briefly, ovarian cancer patients aged 18–79 years at diagnosis who were capable of completing the questionnaire were eligible to participate. A control series was selected at random from the electoral roll (enrolment to vote is compulsory in Australia) frequency matched to cases on age (20-year bands) and broad geographic region. Women with a history of ovarian cancer or bilateral oophorectomy, were not eligible to be controls; cases not on the electoral roll were excluded from the analysis.

Identically trained interviewers administered a standard questionnaire to each woman in a face-to-face interview to obtain personal details including education, height and weight, smoking history, family history of ovarian and other cancers, full reproductive and contraceptive histories and age at menopause. Questions were also asked about tubal sterilization and hysterectomy, and with the women's written consent, confirmation of these procedures was sought from relevant medical practitioners as described previously (Green et al, 1997*a*).

Information was gathered about use of HRT, specifically the type of hormones, doses, age at first use and duration of use were recorded for up to three different types of treatment. Any HRT used by cases after diagnosis was not considered; and those using HRT at diagnosis were considered to be current users. HRT use in controls was treated similarly.

Relative risks of ovarian cancer, both overall and within subcategories, were estimated using multiple logistic regression to simultaneously adjust for age (in years), level of education, residential location, parity, duration of oral contraceptive pill use, talc use in the perineal region, body mass index (BMI), smoking status, hysterectomy and tubal sterilization (where appropriate) and a family history of breast or ovarian cancer (Purdie et al, 1995). Women who had ever used unopposed ORT for at least 1 month were compared with those who had never used HRT, so women who had used other forms of HRT (63 cases and 74 controls) were excluded from the unopposed oestrogen analyses. Similar analyses were performed among women with and without a history of tubal sterilization or hysterectomy to assess potential effect modification from these procedures. Interactions between factors were assessed using the product term in the logistic regression models. All analyses were carried out using the SAS statistical package.

### RESULTS

There were 793 eligible cases and 855 eligible controls in the study representing response rates of 90% and 73% respectively. Ovarian cancer was subdivided into five histological groups: serous (415, 52%); endometrioid and the related clear cell (164, 21%); mucinous (114, 14%); poorly differentiated adenocarcinomas (54, 7%); and mixed epithelial tumours with malignant mixed mesodermal/Müllerian tumours (44, 6%). Two cases with transitional cell (malignant Brenner) tumours were not included in the subtype analyses.

Overall, 131 cases (16.5%) and 149 controls (17.4%) reported ever taking any form of HRT for menopause-related reasons, which is consistent with cross-sectional data from a similar aged population-based Australian study (Dennerstein et al, 1994). Of these, 68 cases (8.6%) and 75 controls (8.8%) reported ever using unopposed ORT. After adjustment for potential confounders, there were no significant associations found between occurrence of ovarian cancer and ever use of HRT, either overall or in its various forms (Table 1). There were also no clear trends in risk for increased duration of HRT use or time since last use, either overall or for the most common forms (Table 2).

There was, however, higher use of ORT among cases with endometrioid or clear cell cancers than among controls or other cases and, after adjustment for potential confounders, a marked and significant elevation in risk among this group of cases was observed (Table 3). A comparison of the effect in endometrioid or clear cell tumours with the effect in other tumour types approached significance ($P$ = 0.06). The separate risks for endometrioid and for clear cell tumours were nearly identical.

Women who reported having either a hysterectomy or tubal sterilization had higher usage of HRT, in particular unopposed oestrogen: 30% of women with a hysterectomy and 15% of women with a tubal sterilization had ever used ORT, compared with only 6% of women without such surgery. These women were also at lower risk of ovarian cancer (Green et al, 1997*b*). We therefore examined whether hysterectomy or tubal sterilization, as well as being confounders, modified the relationship between ever use of ORT and ovarian cancer risk. For all cases, an inverse association between unopposed oestrogen use and ovarian cancer was seen in women with a prior hysterectomy, and no relationship was seen in those with tubal sterilization alone. In contrast, women who reported neither hysterectomy nor tubal sterilization exhibited a moderately strong and significant increase in risk associated with ORT (Table 4). The interaction effects between hysterectomy and ORT and between tubal sterilization and ORT among those women without a hysterectomy were significant ($P$ = 0.006 and 0.04 respectively). Relative risks of unopposed oestrogen therapy were also higher for endometrioid and clear cell tumours than for other tumours among women with a prior hysterectomy or tubal sterilization and strikingly so among those without surgery (Table 4).

 *© 1999 Cancer Research Campaign*

**Table 2** Duration of use and time since last use of hormone replacement therapy, overall and specifically for unopposed oestrogen and oestrogen in combination with progestogen, among all cases of epithelial ovarian cancer and controls with crude and adjusted odds ratios

| HRT use | Cases (*n* = 793) | Controls (*n* = 855) | Crude OR (95% CI) | Adjusted OR[a] (95% CI) |
|---|---|---|---|---|
| Never used[b] | 83.5% | 82.6% | 1.00 | 1.00 |
| Used any HRT for[c] | | | | |
| <1 year | 3.5% | 5.1% | 0.68 (0.42–1.10) | 0.70 (0.41–1.19) |
| 1–3 years | 5.3% | 4.9% | 1.07 (0.69–1.66) | 1.11 (0.88–1.41) |
| >3 years | 6.3% | 6.3% | 0.99 (0.66–1.47) | 1.13 (0.91–1.41) |
| Last used any HRT | | | | |
| Current users | 8.6% | 11.0% | 0.77 (0.55–1.07) | 0.95 (0.67–1.36) |
| 1–5 years ago | 3.5% | 2.7% | 1.30 (0.74–2.28) | 1.28 (0.95–1.72) |
| >5 years ago | 3.0% | 2.7% | 1.11 (0.62–1.99) | 1.03 (0.75–1.42) |
| Used unopposed oestrogen for | | | | |
| < 1 year | 2.4% | 2.6% | 0.92 (0.49–1.72) | 0.93 (0.47–1.85) |
| 1–3 years | 3.4% | 3.0% | 1.11 (0.64–1.92) | 1.13 (0.85–1.50) |
| >3 years | 2.7% | 3.2% | 0.83 (0.46–1.48) | 0.92 (0.67–1.25) |
| Last used unopposed oestrogen | | | | |
| Current users | 3.5% | 4.3% | 0.81 (0.49–1.33) | 0.94 (0.56–1.60) |
| 1–5 years ago | 2.3% | 1.8% | 1.28 (0.64–2.56) | 1.19 (0.82–1.72) |
| >5 years ago | 2.6% | 2.7% | 0.97 (0.53–1.78) | 0.94 (0.67–1.30) |
| Used oestrogen and progestogen in combination for | | | | |
| <1 year | 1.1% | 1.6% | 0.69 (0.29–1.59) | 0.80 (0.33–1.97) |
| 1–3 years | 1.9% | 1.6% | 1.14 (0.55–2.39) | 1.36 (0.92–2.00) |
| >3 years | 1.8% | 1.6% | 1.07 (0.50–2.25) | 1.33 (0.88–2.00) |
| Last used oestrogen and progestogen in combination | | | | |
| Current users | 3.9% | 4.6% | 0.85 (0.52–1.37) | 1.24 (0.73–2.09) |
| Past users | 0.9% | 0.4% | 2.49 (0.64–9.66) | 2.02 (0.99–4.14) |

[a]ORs adjusted for age, education, area of residence, BMI, hysterectomy, tubal sterilization, talc use in perineal region, smoking status, duration of OCP use, parity and a family history of breast or ovarian cancer. [b]Reference category for all forms of HRT. [c]Eleven cases and nine controls did not report on duration of HRT use (and hence time since last use could also not be calculated).

**Table 3** Ever use of unopposed oestrogen replacement therapy among women with various histological types of ovarian cancer and among controls

| Histological type | ORT users | Never used HRT[a] | Crude OR (95% CI) | Adjusted OR[b] (95% CI) |
|---|---|---|---|---|
| Controls | 75 (8.8%)[c] | 706 (82.6%) | 1.00 | 1.00 |
| All cancers | 68 (8.6%) | 662 (83.5%) | 0.97 (0.69–1.36) | 1.27 (0.86–1.88) |
| Serous | 36 (8.7%) | 340 (81.9%) | 1.00 (0.66–1.51) | 1.26 (0.78–2.03) |
| Endometrioid/clear cell | 18 (11.0%) | 132 (80.5%) | 1.28 (0.74–2.22) | 2.56 (1.32–4.94) |
| Mucinous | 6 (5.3%) | 106 (93.0%) | 0.53 (0.23–1.25) | 0.85 (0.32–2.23) |
| Undifferentiated | 4 (7.4%) | 45 (83.3%) | 0.84 (0.29–2.39) | 0.51 (0.14–1.89) |
| Mixed epithelial/mesodermal | 4 (9.1%) | 38 (86.4%) | 0.99 (0.34–2.85) | 1.15 (0.35–3.80) |

[a]Excludes women who used another form of HRT (63 cases, 74 controls). [b]ORs adjusted for age, education, area of residence, BMI, hysterectomy, tubal sterilization, talc use in perineal region, smoking status, duration of OCP use, parity and a family history of breast or ovarian cancer. [c]Percentages are based on row totals (including users of other forms of HRT).

The effect of ORT was essentially uniform across the different levels of duration of use, irrespective of surgical history or histological type of tumour. There was, however, a distinct decrease in risk of ovarian cancer seen with increasing time since last use among women with an intact reproductive system. In this group, compared with never-users of HRT, current ORT users were at highest risk (OR 3.92; 95% CI 1.32–11.6); women who had ceased use within the last 5 years were at intermediate risk (OR 2.09; 95% CI 0.90–4.89); and women who had last used oestrogen more than 5 years ago were at lowest risk (OR 1.45; 95% CI 0.90–2.32). No similar trend in risk with time since last use of ORT was apparent in those with a previous hysterectomy or tubal sterilization. Oestrogen doses were too poorly reported by subjects for a useful analysis.

No associations similar to those seen with unopposed oestrogen were found for any other form of HRT.

### DISCUSSION

A non-significant 20% increase in risk of ovarian cancer was found to be associated with ever use of HRT, which is consistent with summary estimates reported in a recently published meta-analysis on the subject (Garg et al, 1998). Ovarian carcinomas are, however, a heterogeneous collection, morphologically, pathogenically and

© 1999 Cancer Research Campaign 

**Table 4** Ever use of unopposed oestrogen replacement therapy in women with and without hysterectomy and tubal sterilization

| Subgroup of women | ORT users | Never used HRT[a] | Crude OR (95% CI) | Adjusted OR[b] (95% CI) |
|---|---|---|---|---|
| *With hysterectomy:* | | | | |
| Controls | 51 (29.8%)[c] | 114 (66.7%) | 1.00 | 1.00 |
| All cases | 22 (19.5%) | 80 (70.8%) | 0.61 (0.35–1.09) | 0.65 (0.34–1.24) |
| Endometrioid/clear cell | 5 (33.3%) | 9 (60.0%) | 1.24 (0.40–3.89) | 1.75 (0.43–7.14) |
| Other cases | 17 (17.4%) | 71 (72.4%) | 0.53 (0.29–1.00) | 0.57 (0.29–1.15) |
| *Without hysterectomy but with tubal sterilization:* | | | | |
| Controls | 9 (5.8%) | 123 (79.3%) | 1.00 | 1.00 |
| All cases | 5 (5.7%) | 74 (84.1%) | 0.92 (0.30–2.86) | 1.03 (0.27–3.96) |
| Endometrioid/clear cell | 2 (12.5%) | 13 (81.3%) | 2.10 (0.41–10.8) | 2.72 (0.27–27.3) |
| Other cases | 3 (4.2%) | 61 (84.7%) | 0.67 (0.18–2.57) | 0.84 (0.17–4.12) |
| *Without hysterectomy or tubal sterilization:* | | | | |
| Controls | 15 (2.8%) | 469 (88.7%) | 1.00 | 1.00 |
| All cases | 41 (6.9%) | 508 (85.8%) | 2.52 (1.38–4.62) | 3.00 (1.54–5.85) |
| Endometrioid/clear cell | 11 (8.3%) | 110 (82.7%) | 3.13 (1.40–7.00) | 4.29 (1.67–11.1) |
| Other cases | 30 (6.5%) | 398 (86.7%) | 2.36 (1.25–4.44) | 2.66 (1.31–5.37) |

[a]Excludes women who used another form of HRT (63 cases, 74 controls). [b]ORs adjusted for age, education, area of residence, BMI, talc use in perineal region, smoking status, duration of OCP use, parity and a family history of breast or ovarian cancer. [c]Percentages are based on row totals (including users of other forms of HRT).

aetiologically (Russell, 1994). Many epidemiological studies have assumed homogeneity for the purposes of studying general aetiological factors. An important outcome has been revealed in the histological subtype analysis of this case–control study, namely, the moderately strong association found between ever use of unopposed oestrogen and endometrioid or, the closely related, clear cell tumours. This parallels the findings of a number of certain other studies that looked specifically at endometrioid ovarian cancers (La Vecchia et al, 1982; Weiss et al, 1982; Cramer et al, 1983; Risch, 1996), although not all (Hartge et al, 1988; Booth et al, 1989; Kaufman et al, 1989; Whittemore et al, 1992; Hempling et al, 1997). Endometrioid and clear cell tumours are broadly regarded as the equivalent of the same cancers in the endometrium (showing proliferative, or ‘pregnancy-related’ secretory changes), whereas serous tumours are neoplastic analogues of the fallopian tube mucosa and mucinous tumours are similar to those of the endocervix or colon (Scully, 1977). An analogy can therefore be drawn between our findings and those in endometrial cancer in which the relationship with oestrogens is most marked for the endometrioid and least strong for serous papillary carcinomas (Bokham, 1983; Smith and McCartney, 1985).

It is clear that the aetiology of endometrial adenocarcinomas is linked to the role of oestrogens (IARC, 1987). That endometrial adenocarcinoma is a ‘generic diagnosis’ and represents a heterogeneous group of neoplasms is also clear, as it is for ovarian cancers (Greven and Corn, 1997).

A finding of interest was that unopposed ORT was inversely associated with all ovarian cancers in women with a prior hysterectomy (although not significantly), but positively associated in women without either hysterectomy or tubal sterilization. In women with tubal sterilization no association was observed. While this is not the first study to report a lower risk of ovarian cancer associated with ORT among women with a hysterectomy (Whittemore et al, 1992; Hartge et al, 1988), others have reported a larger effect of ORT in this group (Booth et al, 1989; Kaufman et al, 1989) or no difference (Risch, 1996). However, no previous study has examined the effect of ORT stratified by hysterectomy and tubal sterilization. The association seen here may imply that non-contraceptive-oestrogen is a risk factor specifically for women with an intact genital tract. In all categories of surgical history, the endometrioid/clear cell group of tumours retained their elevated risk relative to other tumour types. In addition, among women with an intact genital tract, there was also a noticeable decrease in risk with increasing time since last use of unopposed oestrogen, consistent with the type of association with endometrial carcinomas found by some (Green et al, 1996), though not all studies (Paganini-Hill et al, 1989).

The positive links seen with ORT may reflect the suggested role of ovarian endometriosis in the aetiology of endometrioid or clear cell ovarian cancer (Sainz de la Cuesta et al, 1996). Postmenopausal ORT could cause proliferation of the uterine endometrium, and in women with an intact genital tract, could therefore increase the risk of retrograde menstruation (Metzger and Haney, 1989). This, in combination with the increased levels of circulating oestrogens from the oestrogen therapy, could promote ovarian endometriosis (Faulkner and Riemenschneider, 1945). Women without hysterectomy or tubal sterilization who are prescribed unopposed oestrogen may therefore have an increased risk of subsequent ovarian endometriosis and consequently have a higher risk of endometrioid or clear cell ovarian cancer (Sainz de la Cuesta et al, 1996; Paulson, 1997).

Contrary to what might be expected given the well-established association between oestrogen and endometrial adenocarcinoma, unopposed oestrogen was prescribed for some women with an intact uterus in our study until quite recently. Year of first use is, therefore, unlikely to be a major confounding factor. Further, among the 73 women who both used unopposed oestrogen and had a hysterectomy, only three began use of oestrogen before the operation and all three continued use after surgery.

We do not believe that selection or recall biases could have accounted for our finding (Purdie et al, 1995). It is unlikely the results could be explained by incorrect reporting of operations since self-reported histories of hysterectomy and tubal sterilization were checked against available medical reports and 96% and 88% respectively were confirmed (Green et al, 1997*a*). Furthermore, recall of unopposed ORT would hardly differ from other forms of

*© 1999 Cancer Research Campaign*

HRT, which did not show similar effects. However, many of the associations observed in this investigation of HRT were based on small numbers: only 18 cases of endometrioid or clear cell ovarian cancer had ever used unopposed ORT. When further subdivided by history of gynaecological surgery, this problem was even more pronounced.

Prior efforts to evaluate the effects of menopausal oestrogenic agents on ovarian carcinomas as a whole have been inconsistent, some studies showing an increase in risk (Weiss et al, 1982) some a decrease (Hartge et al, 1988; Hempling et al, 1997) and others no significant effect (La Vecchia et al, 1982; Kaufman et al, 1989; Purdie et al, 1995). These inconsistencies may be due to the histological type-specific associations observed in these data, as well as to the effect modification observed with certain genital tract surgery. Those studies that examined the effect of menopausal oestrogen separately for the different types of ovarian cancer have also yielded contradictory findings. Those not finding a positive association more often used hospital based controls (Hartge et al, 1988; Booth et al, 1989; Kaufman et al, 1989; Hempling et al, 1997), which may reflect an excess of HRT use among hospitalized women (Rodriguez et al, 1995).

In conclusion, our results are consistent with the possibility that some utero-ovarian carcinogenic influence, such as endometriosis, is promoted by ORT, increasing the risk of endometrioid and clear cell ovarian carcinomas.

### ACKNOWLEDGEMENTS

Funding for this study was provided by the Australian National Health and Medical Research Council and the Queensland Cancer Fund. We acknowledge the support and assistance of the Survey of Women's Health study research group, namely the following collaborators: Sèamus Campbell, Chris Dalrymple, Arthur Day, Alan Ferrier, Keith Free (deceased), Peter Grant, Paul Harnett, Phil Harvey, Roger Houghton, Tom Jobling, Peter MacCullum Cancer Institute, Robert Planner, Tony Proietto, Robert Rome, John Solomon, Beatrice Susil, Gerry Wain, Gordon Wright; and research assistants: Carol Birks, Patrica Brisley, Paula Candlish, Suzanne Clarke, Lorraine Dommett, Kerri-Ann Lockwood, Lauretta Luck, Helen Merry, Teresa Pangan, Roslyn Patterson, Louise Potter, Ann Ward, Mignon Watson, Mary-Ellen Yarker. Thanks, of course, also to the doctors who allowed us to interview their patients and to all the women who participated in the study.

### REFERENCES

Bokhman JV (1983) Two pathogenetic types of endometrial carcinoma. *Gynecol Oncol* **15**: 10

Booth M, Beral V and Smith P (1989) Risk factors for ovarian cancer: a case–control study. *Br J Cancer* **60**: 592–598

Cramer DW, Hutchinson GB, Welch WR, Scully RE and Ryan KJ (1983) Determinants of ovarian cancer risk. I. Reproductive experiences and family history. *J Natl Cancer Inst* **71**: 711–716

Dennerstein L, Shelly J, Smith AMA and Ryan M (1994) Hysterectomy experience among mid-aged Australian women. *Med J Aust* **161**: 311–313

Faulkner RL and Riemenschneider EA (1945) Reactivation of endometriosis by stilbestrol therapy. *Am J Obstet Gynecol* **105**: 560

Garg PP, Kerlikowske K, Subak L and Grady D (1998) Hormone replacement therapy and risk of epithelial ovarian carcinoma: a meta-analysis. *Obstet Gynecol* **92**: 472–479

Grady D, Gebretsadik T, Kerlikowske K, Ernster V and Petitti D (1995) Hormone replacement therapy and endometrial cancer risk: a meta-analysis. *Obstet Gynecol* **85**: 304–313

Green A, Purdie D, Green L, Dick M-L, Bain C and Siskind V (1997*a*) Validity of self-reported hysterectomy and tubal sterilisation. *Aust N Z J Public Health* **21**: 337–340

Green A, Purdie D, Bain C, Siskind V, Russell P, Quinn M and Ward B (1997*b*) Tubal sterilisation, hysterectomy and decreased risk of ovarian cancer. *Int J Cancer* **71**: 948–951

Green PK, Weiss NS, McKnight B, Voigt LF and Beresford SAA (1996) Risk of endometrial cancer following cessation of menopausal hormone use (Washington, United States). *Cancer Causes Control* **7**: 575–580

Greven KM and Corn BW (1997) Endometrial cancer. *Curr Probl Cancer* **21**: 65–127

Hartge P, Hoover R, McGowan L, Lesher L and Norris HJ (1988) Menopause and ovarian cancer. *Am J Epidemiol* **127**: 990–998

Hempling RE, Wong C, Piver MS, Natarajan N and Mettlin CJ (1997) Hormone replacement therapy as a risk factor for epithelial ovarian cancer: results of a case-control study. *Obstet Gynecol* **89**: 1012–1016

International Agency for Research on Cancer (1987) *Overall Evaluations of Carcinogenicity: an Updating of IARC Monographs*, Vol 1–42. *IARC Monogr Eval Carcinog Risk Chem Hum* (Suppl 7), pp 280–283. IARC: Lyon

Kaufman DW, Kelly JP, Welch WR, Rosenberg L, Stolley PD, Warshauer ME, Lewis J, Woodruff J and Shapiro S (1989) Noncontraceptive estrogen use and epithelial ovarian cancer. *Am J Epidemiol* **130**: 1142–1151

La Vecchia C, Liberati A and Franceschi S (1982) Noncontraceptive estrogen use and the occurrence of ovarian cancer. *J Natl Cancer Inst* **69**: 1207

Metzger DA and Haney AF (1989) Etiology of endometriosis. *Obstet Gynecol Clin North Am* **16**: 1–14

Paganini-Hill A, Ross RK and Henderson BE (1989) Endometrial cancer and patterns of use of oestrogen replacement therapy: a cohort study. *Br J Cancer* **59**: 445–447

Paulson RJ (1997) Fertility drugs and ovarian epithelial cancer: the endometriosis hypothesis. *J Assist Reprod Genet* **14**: 228–230

Purdie D, Green A, Bain C, Siskind V, Ward B, Hacker N, Quinn M, Wright G, Russell P and Susil B (1995) Reproductive and other factors and risk of epithelial overian cancer: an Australian case-control study. *Int J Cancer* **62**: 678–684

Risch HA (1996) Estrogen replacement therapy and risk of epithelial ovarian cancer. *Gynecol Oncol* **63**: 254–257

Rodriguez C, Calle EE, Coates RJ, Miracle-McMahill HL, Thun MJ and Health CW (1995) Estrogen replacement therapy and fatal ovarian cancer. *Am J Epidemiol* **141**: 828–835

Russell P (1994) Surface epithelial-stromal tumors of the ovary. In: *Blaustein's Pathology of the Female Genital Tract*, 4th edn, Kurman RJ (ed), pp. 705–782. Springer-Verlag: New York

Sainz de la Cuesta R, Eichhorn JH, Rice LW, Fuller AF Jr, Nikrui N and Goff BA (1996) Histologic transformation of benign endometriosis to early epithelial ovarian cancer. *Gynecol Oncol* **60**: 238–244

Scully RE (1977) Ovarian tumors. *Am J Pathol* **87**: 686–720

Smith M and McCartney AJ (1985) Occult, high-risk endometrial cancer. *Gynecol Oncol* **22**: 154

Weiss NS, Lyon JL, Krishnamurthy S, Dietert SE, Liff JM and Daling JR (1982) Noncontraceptive estrogen use and the occurrence of ovarian cancer. *J Natl Cancer Inst* **68**: 95–98

Whittemore AS, Harris R, Itnyre J and The Collaborative Ovarian Cancer Group (1992) Characteristics relating to ovarian cancer risk: collaborative analysis of 12 US case-control studies. II. Invasive epithelial ovarian cancers in white women. *Am J Epidemiol* **136**: 1184–1203

© 1999 Cancer Research Campaign
